# Role of differentially expressed microRNA-139-5p in the regulation of phenotypic internal anal sphincter smooth muscle tone

**DOI:** 10.1038/s41598-017-01550-5

**Published:** 2017-05-03

**Authors:** Jagmohan Singh, Ipsita Mohanty, Sankar Addya, Benjamin Phillips, Hwan Mee Yong, Steven S. An, Raymond B. Penn, Satish Rattan

**Affiliations:** 1 Department of Medicine, Division of Gastroenterology & Hepatology, Sidney Kimmel Medical College of Thomas Jefferson University, Philadelphia, PA USA; 2 Kimmel Cancer Center, Thomas Jefferson University, Philadelphia, PA USA; 3 Department of Surgery, Division of Colorectal Surgery, Thomas Jefferson University, Philadelphia, PA USA; 4 Department of Environmental Health and Engineering, Johns Hopkins Bloomberg School of Public Health, Baltimore, Maryland USA; 5 Center for Translational Medicine (RP), Sidney Kimmel Medical College of Thomas Jefferson University, Philadelphia, Pennsylvania USA

## Abstract

The present study focused on the role of microRNA-139-5p (miRNA-139-5p) in the regulation of basal tone in internal anal sphincter (IAS). Applying genome-wide miRNA microarrays on the phenotypically distinct smooth muscle cells (SMCs) within the rat anorectrum, we identified miRNA-139-5p as differentially expressed RNA repressor with highest expression in the purely phasic smooth muscle of anococcygeus (ASM) vs. the truly tonic smooth muscle of IAS. This pattern of miRNA-139-5p expression, previously shown to target ROCK2, was validated by target prediction using ingenuity pathway (IPA) and by qPCR analyses. Immunoblotting, immunocytochemistry (ICC), and functional assays using IAS tissues and cells subjected to overexpression/knockdown of miRNA-139-5p confirmed the inverse relationship between miRNA-139-5p and ROCK2 expressions/IAS tone. Overexpression of miRNA-139-5p caused a decrease, while knockdown by anti-miRNA-139-5p caused an increase in the IAS tone; these tissue contractile responses were confirmed by single-cell contraction using magnetic twisting cytometry (MTC). These findings suggest miRNA-139-5p is capable of significantly influencing the phenotypic tonicity in smooth muscle via ROCK2: a lack of tone in ASM may be associated with the suppression of ROCK2 by high expression of miRNA-139-5p, whereas basal IAS tone may be associated with the persistence of ROCK2 due to low expression of miRNA-139-5p.

## Introduction

There is widespread agreement that basal tone in the internal anal sphincter (IAS) plays an important role in a number of rectoanal motility disorders such as rectoanal incontinence^1–5^. There is an agreement as well that unique myogenic properties of the smooth muscle cells (SMCs) in the IAS are primarily responsible for the basal tone^6–13^. However, molecular mechanisms underlying the IAS tone are not well understood. In this regard, humans as well as animal studies have shown that constitutively active and upregulated RhoA/ROCK is primarily responsible for the tone^11–21^. RhoA/ROCK leads to phosphorylation of myosin-binding subunit of myosin light-chain phosphatase (MLCP) (p-MYPT1). The latter by inhibiting MLCP leads to increased phosphorylation of the regulatory myosin light-chain (p-MLC_20_) which promotes smooth muscle tone^19, 22, 23^.

To further delineate the role of RhoA/ROCK in the genesis of IAS tone, systematic side-by-side functional and molecular studies have been performed to compare adjoining smooth muscles within the rat anorectum. The IAS by virtue of maintaining spontaneous tone is considered truly tonic and differs from rectal smooth muscle (RSM) which is semi tonic as it has a mixture of tonic and phasic activities. In contrast, smooth muscle of anococcygeus (ASM) has no tone, contracts briefly only following a stimulus, and is purely phasic^11, 18, 24, 25^. Numerous studies have identified an important gradient in the expression levels of RhoA/ROCK in direct correlation with the phenotypic tonicity in these smooth muscles^11, 18, 24, 25^. However, the underlying molecular mechanisms for the high expression of RhoA/ROCK in IAS vs. other smooth muscles are not known.

MicroRNAs (miRNAs) generally considered to be RNA repressors, play an important role in evolution and maintenance of the phenotypic characteristics of gastrointestinal tract smooth muscle by controlling the expression of smooth muscle-specific genes such as Kruppel like factor (KLF4), myocardin and serum response factor (SRF)^26–28^. Likewise, studies in rat IAS and mouse stomach have shown that miRNA-133a downregulates RhoA in aging- and diabetes-associated decrease in the respective smooth muscles’ contractility^29, 30^. It is well known that, in the smooth muscle contractility, ROCK, specifically ROCK2 is an important downstream target of RhoA and is expressed markedly higher in the tonic tissues such as the IAS^11, 12, 31–33^. However, to date there are no data to discern molecular mechanism(s) underlying the differential expression of ROCK2 in determining fundamental molecular differences between the tonic vs. the phasic smooth muscle phenotypes.

Considering the importance of miRNAs in regulating the expression of a number of genes in the smooth muscle^26, 27, 29, 34^, it was considered important to determine the role of miRNAs in the tonic vs. phasic smooth muscle. Important insight into this question was provided by miRNA microarray analyses that identified miRNA-139–5p to be one of the most differentially expressed miRNAs in three phenotypic smooth muscles with a gradient of negative correlation with the levels of tone. Further literature and network analysis revealed that in different systems, miRNA-139-5p targets ROCK2^35, 36^.

The main objective of the present study was to determine the role of miRNA-139-5p in the IAS tone in relation to ROCK2. We first performed differential miRNA microarray analysis using diverse phenotypic smooth muscles: purely tonic (IAS), semi tonic (RSM), and purely phasic (ASM) smooth muscle. This comparison revealed a gradient of expression of miRNA-139-5p with ASM > RSM > IAS, with miRNA-139-5p expression negatively correlating with the known levels of tone in the tissues. We subsequently performed miRNA-139-5p gain- and loss- of function studies to define the role of miRNA-139-5p in the ROCK2-dependent regulation of smooth muscle phenotype. Our findings significantly advance current insight into the molecular regulation of the IAS tone, with direct implications in a number of gastrointestinal and other systemic smooth muscle disorders.

## Materials and Methods

### Animals

Adult Sprague Dawley rats of 6 months age were used for the study and the experimental protocols were approved by the Institutional Animal Care and Use Committee (IACUC) of Thomas Jefferson University.

### Isolation of SMCs

SMCs from IAS, RSM and ASM were isolated as per our previous studies^37^. Briefly, tissues were cut into 2 mm cubes and incubated in oxygenated Krebs physiological solution (KPS) containing 0.1% collagenase and 0.01% trypsin inhibitor at 37 °C for 3 h. During incubation the buffer was replenished thrice after 1 h each, and at the end cells were centrifuged at 350 g for 10 min, followed by washing with fresh oxygenated KPS without enzymes. Thereafter, mixture was centrifuged at 350 g for 10 min at room temperature and the cells in pellet were resuspended at the density of 3 × 10^4^ cells/ml on collagen-coated plates in DMEM with 5% fetal bovine serum, 5% penicillin streptomycin, 50 µg/ml gentamicin, 0.2 µg/ml amphotericin B, and 50 µg/ml sodium ascorbate in 100-mm tissue culture dishes (Corning) at 37 °C and 5% CO_2_ in an incubator with regulated humidity.

### MicroRNA microarrays

miRNA expression studies for IAS, RSM and ASM SMCs were performed as described previously^29, 38, 39^. Briefly, miRNA fractions were isolated from the purified SMCs from the IAS, RSM and ASM, by using miRVana miRNA Isolation kit following the manufacturer’s protocol (Life Technologies-Thermo Fisher Scientific). Affymetrix GeneChip miRNA-1.0 Arrays (Affymetrix, Santa Clara, CA) were hybridized with Flash Tag biotin-labeled total RNA (500 ng) from samples in 100 µl hybridization cocktail. Target denaturation was performed at 99 °C for 5 min, and then 45 °C for 5 min, followed by hybridization at 48 °C for 18 h. Arrays were washed and stained using Fluidic Station 450, and hybridization signals were amplified using antibody amplification with goat IgG and anti-streptavidin biotinylated antibody, followed by scanning via Affymetrix Gene Chip Scanner 3000 using Command Console Software. miRNA data were analyzed by Affymetrix MiRNA QC tool and Genespring V 14.5 software (Agilent, Palo Alto, CA) using Robust Multichip Average. These studies were performed at Cancer Genomics Laboratory of the Thomas Jefferson University. Heat map was generated from the list of differentially expressed miRNAs, which was loaded into Ingenuity Pathway Analysis (IPA) 8.0 software (Ingenuity Systems, Redwood City, CA) (http://www.ingenuity.com) for biological network and functional analyses.

### Validation of differentially regulated miRNA from genome-wide microarray by quantitative real-time PCR (qPCR)

RNA samples (n = 4 rats) were used for each set of SMCs. miRNA RT-PCR was performed using miR cDNA Synthesis Kit and SYBR Green master mix RT-PCR Kit (Promega, Madison, WI). Specific primers to rat miRNAs were designed and synthesized by Qiagen (Qiagen Inc., Valencia, CA). qPCR Ct values were plotted using GraphPad Prism software and results were normalized against U6B RNA.

### Transfection of miRNA and anti-miRNA-139-5p

*In vitro* transfections of locked nucleic acid (LNA) miRNA-139-5p and anti-miRNA-139-5p (Exiqon, MA) in SMCs and tissue strips were performed by using Hiperfect transfection reagent from Qiagen. 100 nM miRNA or 100 nM anti-miRNA was mixed with transfection reagent in serum free DMEM and added to SMCs and tissue strips. SMCs were incubated for 72 h and tissue strips for 48 h, to study the proteomic and functional changes respectively.

### Western blot (WB) analysis

The IAS SMCs and tissue lysates were subjected to WB analysis for protein expression analysis as described previously^13^. Briefly, total protein from each sample was separated by sodium dodecyl sulfate-polyacrylamide gel electrophoresis (SDS-PAGE) and transferred to polyvinylidene difluoride membranes (Millipore, Bedford, MA). The membranes were subjected to immunoblot analysis using antibodies from Santa Cruz Biotechnology Inc. (Santa Cruz, CA) and immunoreactivities to proteins relative to GAPDH were determined as described previously^40, 41^.

### Immunocytochemical (ICC) analysis

Using IAS SMCs, ICC was performed at the basal state, for RhoA, ROCK2, p-MYPT1 and p-MLC_20_ as described previously^13^. The nuclei were stained with DAPI. The images were captured as single acquisitions using Nikon 80i microscope. Images were imported into Image J (National Institutes of Health) for quantification. Average fluorescence intensity from single cell was calculated by randomly selecting five different areas of 5 μm^2^ around the cytoplasm, excluding nuclear area. These values were averaged and used as n = 1. The above process was repeated for 40 cells at random and calculated as means ± s.e.m. To get intensity per unit area average intensity was divided by the total area selected for calculating the intensity. Texas red-conjugated IgGs from mouse, goat and rabbit were used as background fluorescence intensity controls.

### Force measurement

The smooth muscle strips (~1 × 10 mm) prepared from the circular smooth muscle layer of the IAS were used for the force experiments as described previously^41^. Strips were transfected with miRNA-139-5p as per^29^. Briefly, the IAS tissue strips were transferred into 2-ml muscle baths containing KPS that was continuously perfused with carbogen (95% O_2_ + 5% CO_2_). The composition of KPS was as follows (mM): 118.07 NaCl, 4.69 KCl, 2.52 CaCl_2_, 1.16 MgSO_4_, 1.01 NaH_2_PO_4_, 25 NaHCO_3_, and 11.1 glucose. The isometric force was recorded using force transducers (FORT10, WPI, 108 Sarasota, FL). Initially, the strips were stretched using 1.0 g of tension, after which they were allowed to equilibrate for at least 60 min, during which time they were repeatedly washed with fresh KPS every 20 min. All force data were monitored using Chart 4.1.2 via a PowerLab/8SP data-acquisition system (ADInstruments, Colorado Springs, CO)^41^. The spontaneously developed basal IAS tone, and its maximal increase and decrease were calculated with reference to the responses to bethanechol (100 µM), and 0 Ca^2+^ respectively, in the beginning and at the end of each experiment. Concentration-response curves (CRC) for thromboxane A_2_ analog U46619 (1 nM to 1 µM), and single concentration contractile response for U46619 (1 µM) were examined in the muscle strips pretreated for 48 h with scrambled miRNA (control), and miRNA-139-5p before and after anti-miRNA-139-5p.

### Magnetic twisting cytometry (MTC)

Individual SMCs contraction was measured directly using MTC, which monitors dynamic changes in cell stiffness as described previously^42^. In brief, a functionalized microbead bound to an adherent cell is first magnetized horizontally and then twisted in a vertically aligned homogenous magnetic field (20 Gauss). Here, lateral bead displacement in response to the resulting oscillatory torque is detected via a charge-coupled device camera (Hamamatsu Orca AG) attached to an inverted optical microscope, with an accuracy of 5 nm using an intensity-weighted center-of-mass algorithm^43^. The ratio of specific torque to lateral bead displacements is then taken as a measure of the complex cell stiffness in units of Pascal per nm (Pa/nm). The cell stiffness was measured in each cell and, unless otherwise stated, represented as geometric mean ± s.e.m.^42, 43^. The cells for these studies were obtained from discarded human tissues in accordance with the studies approved by Thomas Jefferson University IRB and based on use of deidentified, discarded tissues were judged to be *Not Human Subjects Research*.

### Statistical analyses

qPCR data for miRNA was replicated in four rats in different experiments. Comparison between 2 groups was made using the 2-tailed Student’s *t* test; and comparison between multiple groups was made using one-way ANOVA using GraphPad Prism 5.0. Data were presented as the mean ± s.e.m.

## Results

### Differential miRNA expression in tonic vs. phasic SMCs

Genome-wide expression profiles via microarray on the miRNA from rat IAS, RSM, ASM SMCs were assessed by employing Affymetrix rat Gene Chip miRNA 1.0 as described previously^29^. The miRNA microarray data were deposited in the Gene Expression Omnibus (GEO) database that are accessible at the following link: http://www.ncbi.nlm.nih.gov/geo/query/acc.cgi?acc=GSE90981.

**Figure 1:**
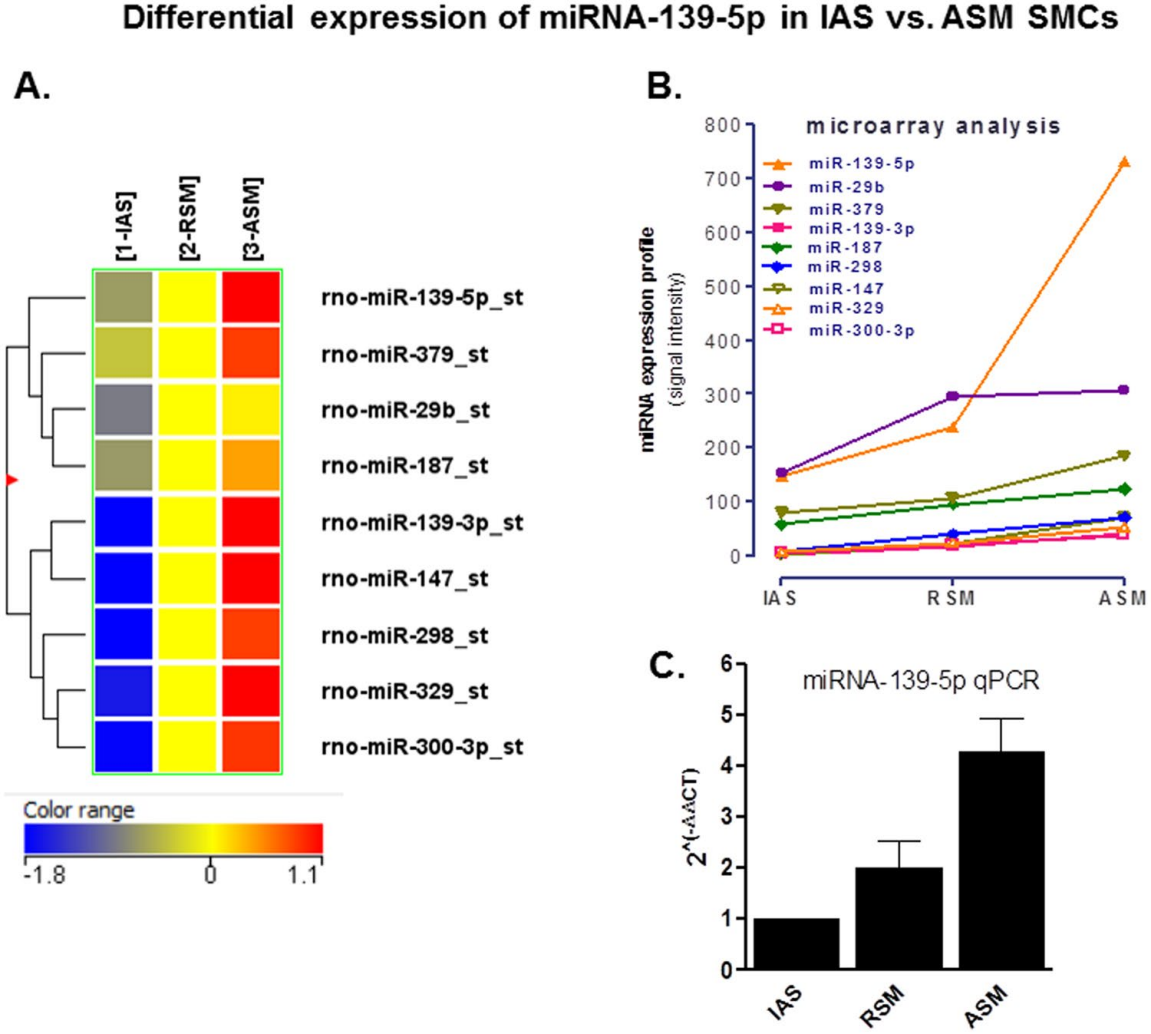
miRNA microarray data and its validation via qPCR. (**A**) Microarray analysis heat map of microRNAs isolated form rat IAS, RSM and ASM SMCs. Nine miRNAs shown in the heat map follow the gradient trend of ASM > RSM > IAS. (**B**) Graph showing that miRNA microarray identified nine differentially expressed miRNAs in an ascending gradient manner from the purely tonic, mixture of tonic and phasic to the purely phasic SMCs as IAS > RSM > ASM. The IAS represents tonic, while RSM and ASM represent semi tonic and purely phasic smooth muscles, respectively. Among nine differentially expressed miRNAs, miRNA-139-5p is the most differentially expressed, with ~4-fold higher expression in the ASM vs. the IAS. (**C**) qPCR analysis graph showing relative expression calculated by using the equation: Δ Ct = Ct miRNA-Ct (RNU6B) and 2^(−ΔΔCT) = 2^(−ΔCt IAS, RSM, ASM−ΔCt IAS) confirms the highest expression of miRNA-139-5p in the ASM vs. the IAS SMCs with pattern similar to that in the differential miRNA microarray (*p < 0.05; n = 4).

Rat IAS (purely tonic) served as control or reference point to determine up- and down-regulation of these gene transcripts in the RSM (a mixture of tonic and phasic) and ASM (purely phasic) smooth muscle phenotypes. Only the miRNAs with noticeable gradient between different SMCs following the trend of ASM > RSM > IAS were presented as a heat map and dendrogram (Fig. 1A). miRNAs showing striking differences between different phenotypic SMCs following the above trend were plotted as a line graph (Fig. 1B). Among different miRNAs, only nine miRNAs met these criteria, and further scrutiny revealed that miRNA-139-5p was the most differentially expressed with highest expression in the ASM > RSM > IAS SMCs. qPCR studies validated this trend of miRNA-139-5p for its significantly differential expression (*p < 0.05; n = 4; Fig. 1C).

### miRNA-139-5p targets in SMCs as shown by IPA

**Figure 2:**
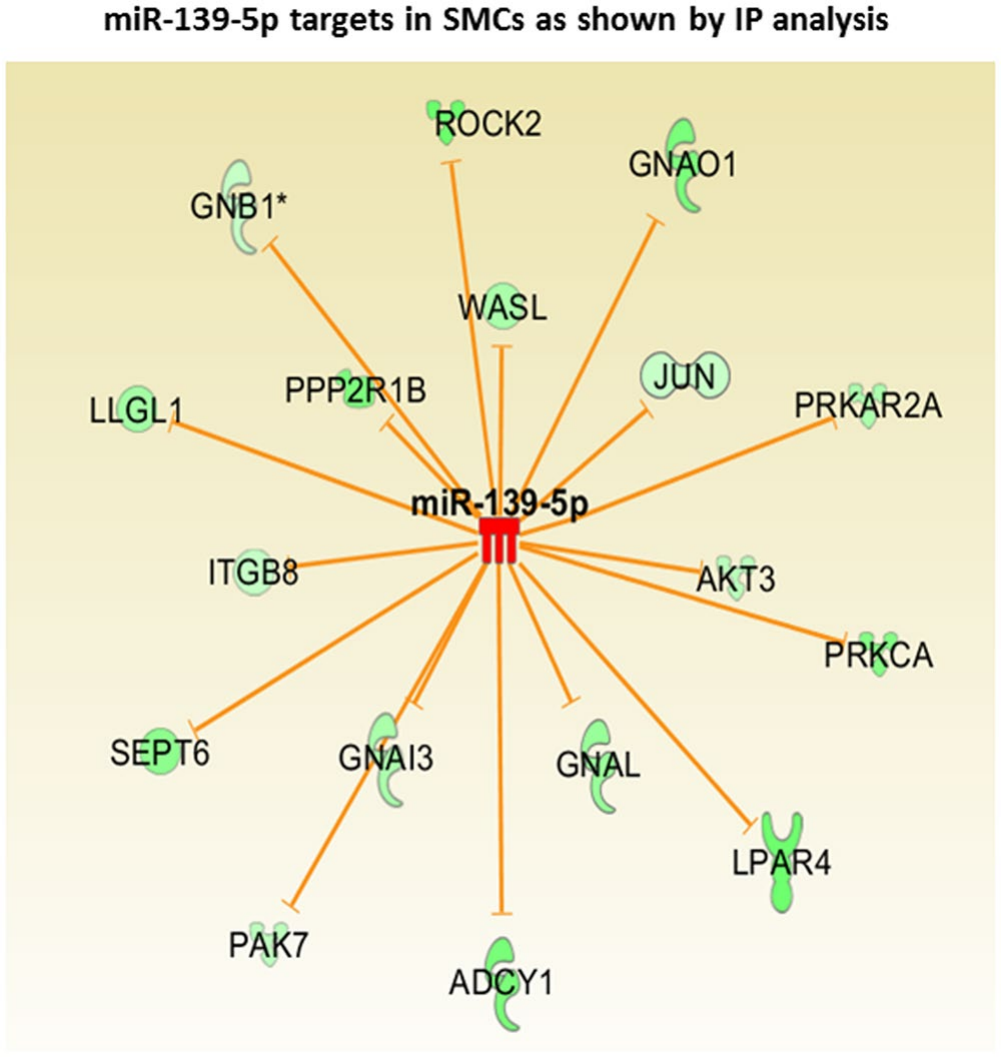
Ingenuity pathway analysis (IPA) showing ROCK2 as one of the direct targets of miRNA-139-5p. miRNA microarray data was subjected to ingenuity pathway analysis (IPA) and various validated targets of miR-139-5p from the literature were plotted as an Interactome. The analysis shows that human ROCK2 is one of the direct targets of miR-139-5p as it has a direct miR-139-5p binding site at its 3′UTR. Some of the other validated targets mentioned in literature, which may also be down-regulated in tonic smooth muscles are given in this interactome.

Possible targets of miRNA-139-5p were identified by IPA analysis as described previously^26, 29^ (Fig. 2). According to previously published studies, one of the direct targets of miRNA-139-5p in humans was identified to be ROCK2^35, 36, 44, 45^. Differential expression of miRNA-139-5p identified above via miRNA microarray and qPCR data (Fig. 1) was further validated using immunoblot and immunofluorescence analyses for Rho kinase and its downstream signaling molecules. To further confirm the functional validity of these data, we monitored smooth muscle force in the IAS and single-cell contractility via MTC. These studies employed the approaches of overexpression and knockdown of miRNA-139-5p using selective oligomer and anti-miRNA-139-5p, respectively.

### Effect of miRNA-139-5p overexpression on RhoA/ROCK pathway

**Figure 3:**
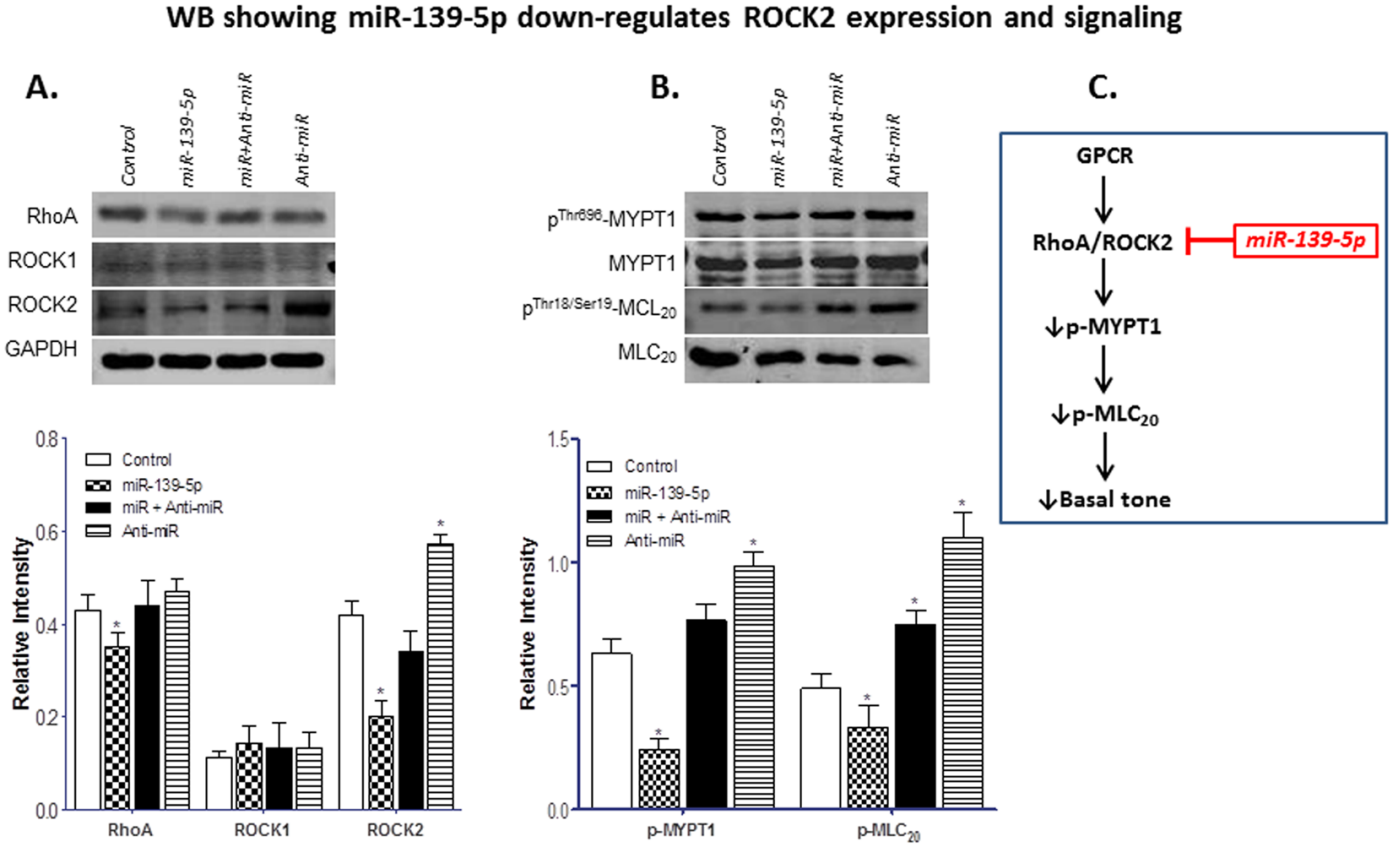
Effect of miRNA-139-5p overexpression on RhoA/ROCK machinery in IAS SMCs. (**A)** Western blot data showing that miRNA-139-5p overexpression in the IAS SMCs decreases the expression of RhoA/ROCK signal transduction machinery proteins which is selectively blocked by the anti-miRNA-139-5p. (**A)** (upper portion). Western blots comparing the expression levels of RhoA, ROCK1, and ROCK2 before and after miRNA-139-5p, anti-miRNA-139-5p alone, and following miRNA-139-5p + anti-miRNA-139-5p. The lower portion of the panel provides the corresponding quantitative data showing that miRNA-139-5p causes significant (*p < 0.05) downregulation of RhoA and ROCK2 without any significant (*p* > 0.05) effect on the levels of ROCK1. Conversely, anti-miRNA-139-5p by itself causes significant upregulation of RhoA and ROCK2 (*p < 0.05). (As indicated, RhoA, ROCK1, and ROCK2 expressions were compared with GAPDH.) (**B)** (upper portion).Western blots comparing the expression levels of p-MYPT1and p-MLC_20_, before and after miRNA-139-5p, anti-miRNA-139-5p alone, and following miRNA-139-5p + anti-miRNA-139-5p. The lower portion of this panel provides the quantitative data showing that miRNA-139-5p significantly (*p < 0.05) downregulates, while anti-miRNA-139-5p causes significant (*p < 0.05) upregulation of p-MYPT1 and p-MLC_20_. (As shown, p-MYPT1, and p-MLC_20_ expressions were compared with MYPT1 and MLC_20_, respectively.) (**C)** Model representing the effect of miRNA-139-5p on RhoA/ROCK2 signaling cascade. The IAS smooth muscle is characterized by upregulated RhoA/ROCK signaling which may be either constitutively active or GPCR-activated. Data suggest that higher levels of miRNA-139-5p in the IAS attenuate ROCK2 expression, which in turn leads to activation of MLCP (via decrease in p-MYPT1), and decrease in p-MLC_20_ and basal tone.

**Figure 4:**
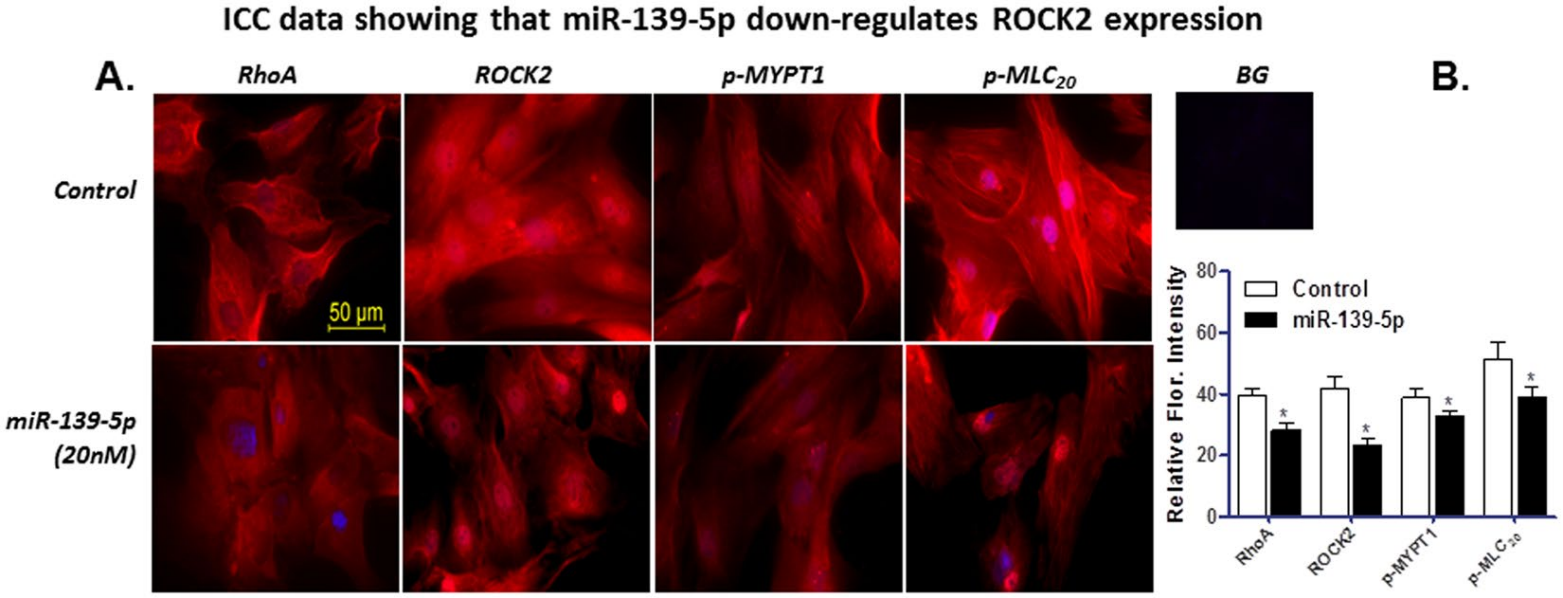
Immunocytochemistry data before and after miRNA-139-5p. (**A**) Immunocytochemical images showing expression of RhoA, ROCK2, p-MYPT1, and p-MLC_20_ following transfection of IAS SMCs with miRNA-139-5p. (**B)** Quantitative data showing significant (*p < 0.05) downregulation of immunofluorescence intensity of RhoA, ROCK2, p-MYPT1, and p-MLC_20_ in the IAS SMCs following transfection with miRNA-139-5p as compared with controls (cells treated with scrambled miRNA). BG = background.

Transfection of rat IAS SMCs with miRNA-139-5p oligonucleotide (for 72 h) caused a significant decrease in the basal expression levels of RhoA/ROCK2, p-MYPT1, and p-MLC_20_ as determined via immunoblot (*p < 0.05; n = 4; Fig. 3A,B). It is noteworthy that, in contrast with the regulation of ROCK2, miRNA-139-5p caused no significant decrease in the expression of ROCK1 (p > 0.05; n = 4), suggesting the specificity of miRNA-139-5p targeting ROCK2. Transfection experiments using immunocytochemical studies followed by immunofluorescence analysis also validated the above notion that miRNA-139-p significantly (*p < 0.05; n = 4; Fig. 4) decreases the expression of RhoA/ROCK2, p-MYPT1, and p-MLC_20_ in the IAS SMCs as compared to the control cells transfected with scrambled miRNA. Figure 3C depicts a model showing miRNA-139-5p associated attenuation of RhoA/ROCK2 signaling leading to decreases in p-MYPT1 and p-MLC_20_.

### Effect of miRNA-139-5p and anti-miRNA-139-5p on the basal tone

**Figure 5:**
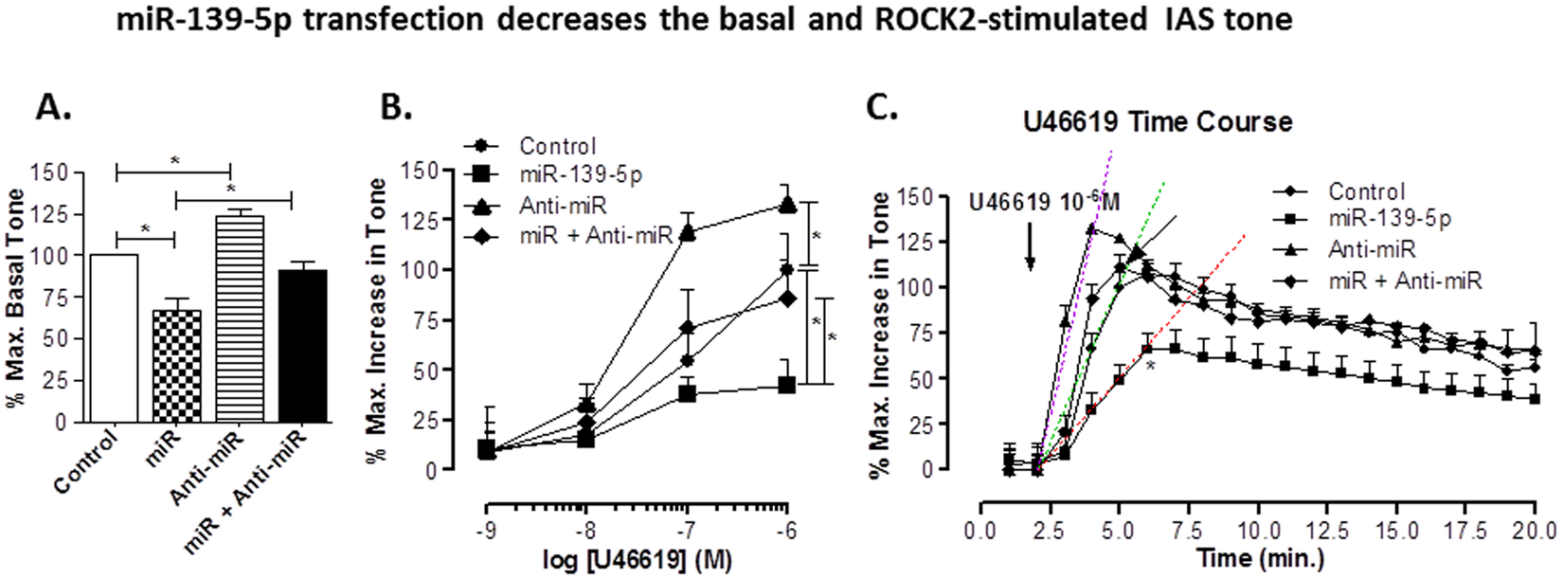
Effect of miRNA- and anti-miRNA-139-5p. (**A**) miRNA-139-5p (100 nM) produces significant (*p < 0.05; n = 5) decrease in the IAS tone, as compared with scrambled miRNA (control), which is significantly blocked by pre-treatment with 100 nM antagomir (*p < 0.05; n = 5). Conversely, 100 nM antagomir by itself significantly (*p < 0.05; n = 4) increases the IAS tone as compared to control. (**B)** miRNA-139-5p significantly shifts U46619 CRC causing an increase in the IAS tone towards right (*p < 0.05; n = 6–8), which is attenuated by the antagomir. In contrast, the antagomir causes a significant shift in the control CRC to left (*p > *0.05; n = 6–8). (**C)** Time course data with U46619 (1 μM) before and after miRNA-139-5p show a significant (*p < 0.05) decrease in the maximal increase in the IAS tone and increase in the time taken to achieve it following miRNA-139-5p transfection. The above described effect of miRNA-139-5p is significantly (*p < 0.05) attenuated by the pre-transfection of the smooth muscles with anti-miRNA-139-5p. Conversely, anti-miRNA-139-5p significantly (*p < 0.05) increases the maximal effect and produces left-ward shift in time-course of U46619-induced contraction. Changes in the shifts in the kinetic velocities following U46619 are shown by the regression analyses with dashed lines.

Considering the role of Rho kinase in maintaining basal IAS SM tone^11, 14, 18^, we investigated whether the decreased expression of ROCK2 by miRNA-139-5p is responsible for the changes in the basal tone. Data showed that transfection of IAS muscle strips with miRNA-139-5p (100 nM for 48 h) significantly decreased (33 ± 7%; p < 0.05; n = 4; Fig. 5A) basal tone. The effect of miRNA-139-5p overexpression was found to be selective given anti-miRNA-139-5p significantly (*p < 0.05; Fig. 5A) reversed the inhibitory effect of miRNA-139-5p on the IAS tone. Of equal significance, in contrast with the effect of miRNA-139-5p, reducing expression of miRNA-139-5p using anti-miRNA-139-5p had the opposite effect, causing an increase in the IAS tone.

### Effect of miRNA-139-5p and anti-miRNA-139-5p on ROCK activator U46619-induced increase in IAS tone

Transfection of the IAS smooth muscle strips with miRNA-139-5p caused a significant rightward shift in the U46619 CRC (*p < 0.05; n = 4; Fig. 5B). Anti-miRNA-139-5p significantly reversed this effect of miRNA-139-5p (*p < 0.05; n = 4). U46619 CRC following the combination of the miRNA and anti-miRNA-139-5p was not significantly different as compared to control (p > 0.05; n = 4; Fig. 5B). These data show the specificity of both miRNA and anti-miRNA-139-5p. Moreover, anti-miRNA-139-5p expression itself caused a significant leftward shift in the U46619 CRC (*p < 0.05; n = 4; Fig. 5B).

### Influence of miRNA-139-5p and anti-miRNA-139-5p on kinetics and amplitude of peak increase in IAS tone by U46619

For these experiments, we determined the time-course effect of miRNA and anti-miRNA-139-5p, alone and in combination, on U46619 (1 µM)-induced increase in the IAS tone over a period of 20 min. Data revealed that miRNA-139-5p inhibited the velocity (shown by the rightward shift in the time-course curve) of fast contraction, and the maximal contraction produced by U46619 (*p < 0.05; n = 4; Fig. 5C). The rightward shift in the velocity following miRNA-139-5p, and its reversal following anti-miRNA was shown by the regression lines. Importantly, ant-miRNA-139-5p had an effect opposite to that of miRNA-139-5p, i.e. it caused a significant increase in the amplitude of maximal contraction with a leftward shift in the U46619 response kinetics during initial phase of contraction (*p < 0.05; n = 4).

### Effect of miRNA-139-5p transfection on the speed of relaxation with 0 Ca^2+^ and of redevelopment of IAS tone following replenishment with normal Ca^2+^

**Figure 6:**
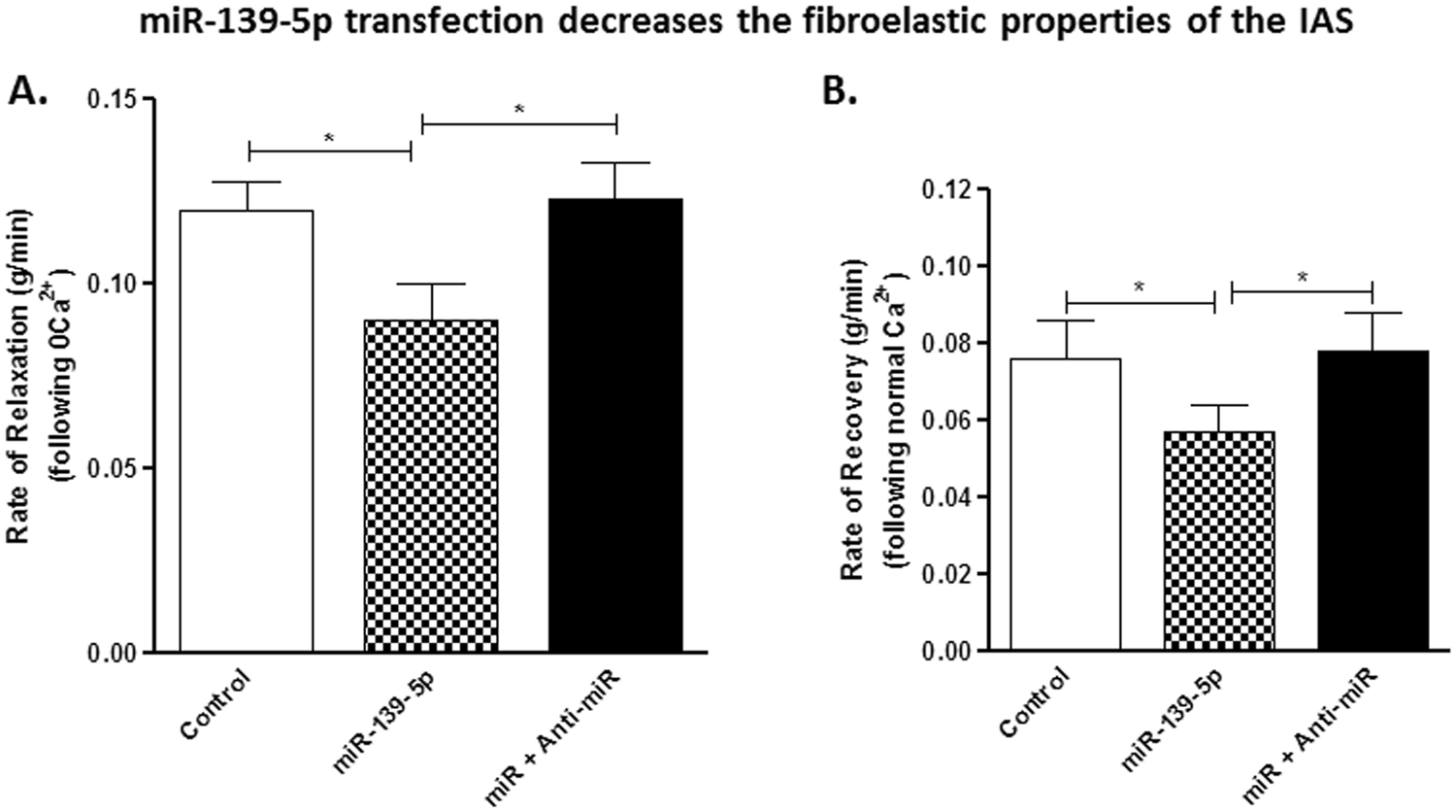
Effect of miRNA- and anti-miRNA-139-5p on IAS kinetics. (**A,B**) Graphs showing rate of relaxation (**A**) and of recovery (**B**) of IAS tone following 0 Ca^2+^ and normal Ca^2+^ replenishment, respectively, following transfection of the smooth muscle strips. miRNA-139-5p significantly (*p < 0.05) decreases the rates of relaxation and of recovery of the IAS tone which are significantly reversed by anti-miRNA-139-5p pre-treatment (*p < 0.05; n = 6–8).

Since RhoA/ROCK plays an important role in the development of IAS tone and in the fibroelastic properties^11, 14, 18, 41, 46^, it was considered important to determine whether miRNA-139-5p by suppressing ROCK2 affects the speed of IAS relaxation and genesis of IAS tone following 0 Ca^2+^, and Ca^2+^ replenishment, respectively. Data revealed that miRNA-139-5p causes a significant decrease in the speed of both relaxation following 0 Ca^2+^ (*p < 0.05; n = 4; Fig. 6A) and of redevelopment of the tone following Ca^2+^ replenishment (*p < 0.05; n = 4; Fig. 6B). Both of these miRNA-139-5p-affected events were significantly reversed by anti-miRNA-139-5p (*p < 0.05). Collectively, these data examining the effect in the basal and ROCK-activated states, and kinetic analyses of the IAS tone suggest that miRNA-139-5p by suppressing ROCK2 expression produces inhibitory effects in the basal tone and on the fibroelastic properties of the smooth muscle.

### Effect of miRNA-139-5p on basal and U46619-induced stiffness using direct IAS SMC contractility assay via MTC

**Figure 7:**
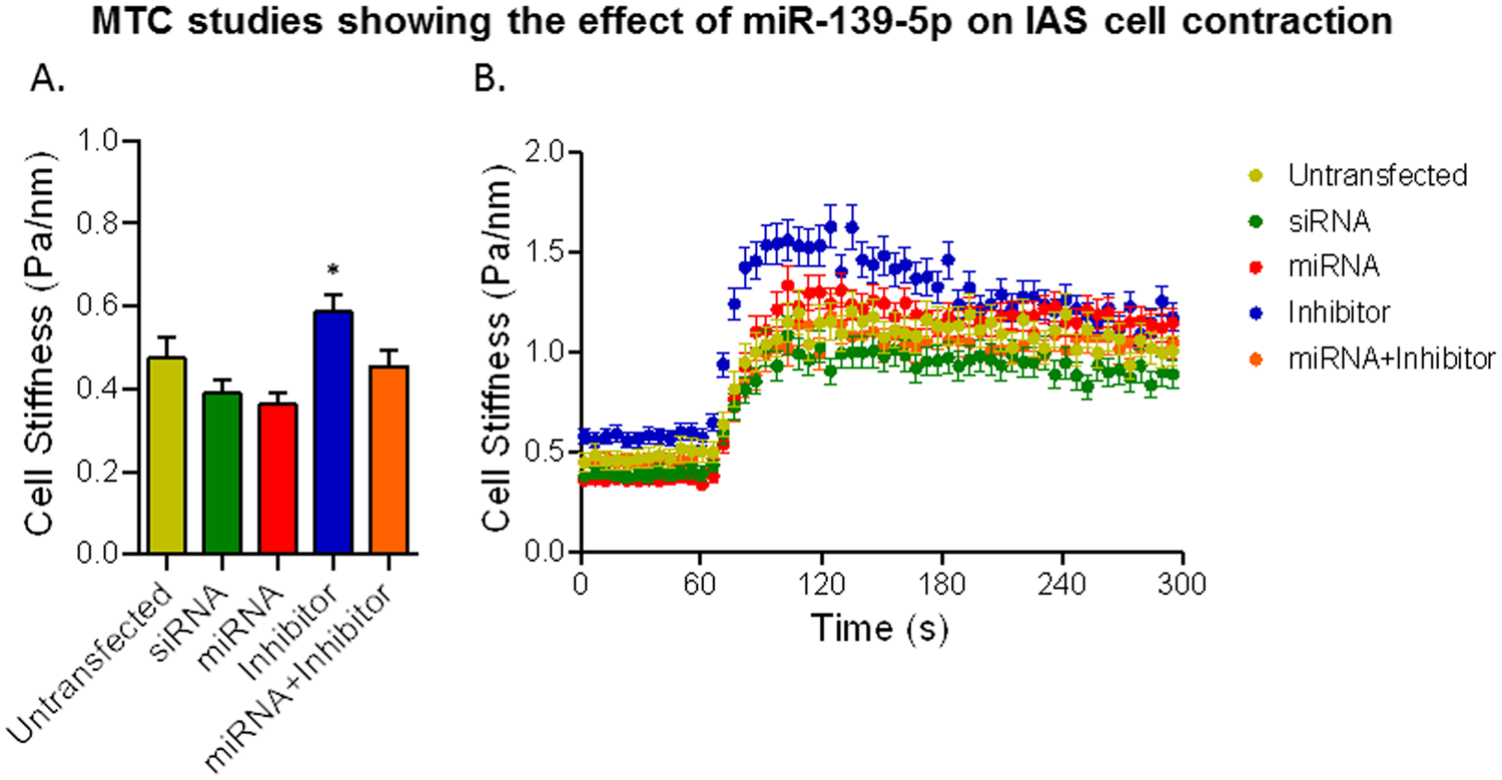
MTC data showing the effect of miRNA- and anti-miRNA-139-5p. (**A**) Baseline cell stiffness of individual IAS SMCs before and after 72 h transfection with ROCK2 siRNA, miRNA-139-5p, anti-miRNA-139-5p (inhibitor), and miRNA-139-5p plus the inhibitor. SMCs transfected with ROCK2 siRNA and miRNA-139-p showed decreases in baseline cell stiffness (basal tone) while cells transfected with anti-miRNA-139-5p showed increases in basal tone. Increased basal tone in response to anti-miRNA-139-5p was attenuated with co-transfection with miRNA-139-5p. Data are presented as Mean ± s.e.m. (n = 89–149 individual cell measurements). (**B)** Dynamic changes the stiffness of IAS SMCs in response to 100 nM U46619. Consistent with the rat smooth muscle data, anti-miRNA-139-5p causes faster and greater cell stiffening in response to U46619.

To further ascertain the mechanism by which miRNA-139-5p regulates the basal tone in IAS, we used MTC and measured dynamic changes in the stiffness of human IAS SMCs as indices of single-cell contraction as described previously^42^. This approach eliminates any potential contamination with other unrecognized mechanisms that may be operative in the IAS smooth muscle preparation. Here, magnetic particles attached to the cytoskeleton of SMCs by a peptide linker provide a highly quantitative measurement of cytoskeletal stiffness of the living cells at the single-cell resolution. Consistent with the data in the smooth muscle strips, MTC data demonstrate that transfection of human IAS SMCs with ROCK2 siRNA and miRNA-139-5p decreased baseline cell stiffness (basal tone) (Fig. 7A). Conversely, transfection of human IAS SMCs with anti-miRNA-139-5p increased basal tone, which was attenuated by co-transfection with miRNA-139-5p (Fig. 7A). Most strikingly, these SMCs transfected with anti-miRNA-139-5p showed faster and greater cell stiffening responses to 100 nM U46619, and this single-cell contraction was inhibited by miRNA-139-5p (Fig. 7B). In addition, since both basal IAS tone^11–14, 16, 18^ and response to U46619^29, 47^, which are critically dependent on RhoA/ROCK activation, were attenuated by ROCK2 siRNA, suggest that miRNA-139-5p exerts its effects via ROCK2 suppression.

## Discussion

The current study examining the phenotypic smooth muscles and cells from IAS (purely tonic), RSM (semi tonic) and the ASM (purely phasic), demonstrate that the expression levels of miRNA-139-5p correlate inversely with those of tone and ROCK2 expression. In addition, these data for the first time show that miRNA-139-5p negatively regulates the tonic smooth muscle function via suppression of RhoA/ROCK2. Previously published studies have reported a significant gradient in the expression levels of RhoA/ROCK highest in the IAS and lowest in the ASM in correlation with the levels of tonicity in the smooth muscle as follows IAS > RSM > ASM^11–14, 16, 18^. Collectively, these data suggest an important role of miRNA-139-5p in the genesis of basal tone in the IAS and lack of tone in the purely phasic smooth muscles such as the ASM.

It is well known that miRNAs suppress the expression of multiple genes via binding to the complimentary sequence to the mRNA. Similar data has been obtained in the gastrointestinal smooth muscle phenotypes both in animals and humans^26–28^. Additionally, miRNAs have been shown to be expressed in a tissue-specific manner, suggesting their role in maintaining smooth muscle-specific genotypic and phenotypic characteristics^34, 48, 49^. Recent studies from our laboratory have shown that aging leads to changes in certain miRNAs such as miRNA-133a that affect IAS tone by targeting RhoA/ROCK signal transduction cascade critical for the basal IAS tone^29^. Similar data were obtained in murine stomach where increase in miRNA-133a was shown to downregulate RhoA and decrease in the smooth muscle contractility^30^. However, the role of miRNAs in the regulation of phenotypic tonic vs. phasic smooth muscles has not been examined before.

Herein, we performed microarray analysis to screen differential expression of miRNAs in the SMCs from IAS, RSM and ASM. Among nine miRNAs, we observed a graded and most differential decrease in miRNA-139-5p expression ASM > RSM > IAS. The findings of microarray differential expression were further confirmed via qPCR analyses. miRNA-139-5p encoded within the second intron of the phosphodiesterase 2 A (PDE2A) gene has been shown to be located on chromosome 11^35^. Of further importance, miRNA-139-5p has been shown to be a potential tumor suppressor in humans by targeting ROCK2^35, 36, 44, 45^.

Targeting of ROCK2 by miRNA-139-5p was further verified by our current IPA as shown in Fig. 2. In addition, present data using qPCR, WB, ICC, and functional data in the IAS tone, and direct contractility assays using MTC, following overexpression and knockdown of miRNA-139-p suggest that miRNA-139-5p negatively regulates the IAS tone via ROCK2.

Our immunoblot and ICC analysis also revealed downregulation of RhoA, ROCK2, pMYPT1, and p-MLC_20_ proteins in miRNA-139-transfected cells, speculatively via targeting ROCK2.

To determine the effect of miRNA-139-5p on smooth muscle function, we overexpressed or knocked down miRNA-139-5p in IAS smooth muscle strips (for force experiments), and SMCs (for MTC experiments monitoring direct contraction assay of SMCs) by transfecting them with synthetic miRNA-139-5p and anti-miRNA-139-5p, respectively.

The smooth muscle transfection experiments with miRNA-139-5p reveal a decrease in the basal tone, and attenuation of ROCK activator U46619-induced an increase in the tone. The blockade of these effects by anti-miRNA-139-5p demonstrates the selectivity of actions of miRNA-139-5p. Of added significance the anti-miRNA-139-5p caused an augmentation in basal tone and its increase following U46619 provide further credence to the hypothesis that miRNA-139-5p plays an important role in the maintenance of tonicity in the IAS via RhoA/ROCK signaling.

Additional experiments show that miRNA-139-5p decreases the speeds of relaxation and recovery to the basal IAS tone following 0 Ca^2+^ and normal Ca^2+^ respectively. Previously published studies have shown that the fibroelastic properties responsible for the speeds of relaxation and recovery of the basal IAS tone are dependent upon RhoA/ROCK machinery^41, 46, 50^. The present study’s MTC experiments complement the above functional studies. The MTC data show that the effects of miRNA-139-5p are similar to those of ROCK2-siRNA in decreasing the basal stiffness of the IAS SMCs and their contractility (measured as maximal increase in amplitude and shifts in the kinetics of initial fast phase of contraction) in response to RhoA/ROCK activator U46619. These data further support the concept that miRNA-139-5p regulates the IAS tone and smooth muscle phenotype differentiation via RhoA/ROCK.

Earlier studies using cancer cell lines^35, 36, 44, 45^ have shown that miRNA-139-5p targets ROCK2 directly in humans. Those data are in agreement with our hypothesis that miRNA-139-5p regulates specific functioning of tonic vs. phasic phenotypic smooth muscles by regulating ROCK2. Since present studies did not determine a direct target site of miR-139-5p in rat ROCK2 UTR previously established in humans, an indirect effect via some other intermediary proteins cannot be ruled out and deserves further investigation. For example, studies in different systems have shown the involvement of IGF-1/insulin-p42/p44MAPKp90RSK-eFF2 signaling axis in the regulation of ROCK2 expression^35, 51^. In addition, involvement of other intermediary pathways or targets of miRNA-139-5p such as TGF-β, Wnt, MAPK, and PI3K reported to inhibit RhoA/ROCK^35^ and microRNA-mediated G protein-coupled receptors (GPCR) crosstalk^52^, require further investigations.

In summary, our studies suggest that miRNA-139-5p plays a significant role in the functional phenotypic smooth muscles by repressing RhoA/ROCK pathway in the SMCs, and that its low expression in the IAS may contribute to the basal tone. Present data suggest an important role of miRNA-139-5p in determining the levels of smooth muscle tone and phenotype differentiation. Such data may lead to new inroads in our understanding of the pathophysiology and therapeutic targeting in the rectoanal motility disorders associated with the IAS tone.
